# Spinal Cord Imaging in Amyotrophic Lateral Sclerosis: Historical Concepts—Novel Techniques

**DOI:** 10.3389/fneur.2019.00350

**Published:** 2019-04-12

**Authors:** Mohamed Mounir El Mendili, Giorgia Querin, Peter Bede, Pierre-François Pradat

**Affiliations:** ^1^Department of Neurology, Icahn School of Medicine at Mount Sinai, New York, NY, United States; ^2^Biomedical Imaging Laboratory (LIB), Sorbonne University, CNRS, INSERM, Paris, France; ^3^Department of Neurology, Pitié-Salpêtrière University Hospital (APHP), Paris, France; ^4^Computational Neuroimaging Group, Trinity College Dublin, Dublin, Ireland

**Keywords:** ALS (Amyotrophic lateral sclerosis), MRI—magnetic resonance imaging, MND, spinal cord, neuroimaging

## Abstract

Amyotrophic lateral sclerosis (ALS) is the most common adult onset motor neuron disease with no effective disease modifying therapies at present. Spinal cord degeneration is a hallmark feature of ALS, highlighted in the earliest descriptions of the disease by Lockhart Clarke and Jean-Martin Charcot. The anterior horns and corticospinal tracts are invariably affected in ALS, but up to recently it has been notoriously challenging to detect and characterize spinal pathology *in vivo*. With recent technological advances, spinal imaging now offers unique opportunities to appraise lower motor neuron degeneration, sensory involvement, metabolic alterations, and interneuron pathology in ALS. Quantitative spinal imaging in ALS has now been used in cross-sectional and longitudinal study designs, applied to presymptomatic mutation carriers, and utilized in machine learning applications. Despite its enormous clinical and academic potential, a number of physiological, technological, and methodological challenges limit the routine use of computational spinal imaging in ALS. In this review, we provide a comprehensive overview of emerging spinal cord imaging methods and discuss their advantages, drawbacks, and biomarker potential in clinical applications, clinical trial settings, monitoring, and prognostic roles.

## Introduction

Amyotrophic lateral sclerosis (ALS) is a relentlessly progressive neurodegenerative disorder. Anterior horn pathology and corticospinal tract degeneration has been identified as a core feature of ALS since the earliest descriptions of the condition (1, 2). Despite repeated attempts to detect and characterize spinal cord pathology *in vivo* (3), technological constraints have traditionally precluded reliable quantitative spinal imaging in ALS. Due to the plethora of methodological challenges, such as the small cross-sectional area of the human spinal cord, respiratory, and cardiac movement effects, the overwhelming majority of imaging studies have focused on cerebral alterations in ALS (4).

The diagnosis of ALS is primarily clinical and requires the careful exclusion of ALS-mimics (5). Given the heterogeneity of clinical presentations and the prevalence of atypical phenotypes, diagnostic delay in ALS is not uncommon, and the average period between symptom onset and definite diagnosis is ~12 months worldwide (6). The median survival from symptom onset ranges from 20 to 48 months (7–9). Progression rates in ALS show considerable variation, and prognosis depends on age at onset, region of onset, co-morbid cognitive impairment, nutritional status, and certain genotypes are associated with faster progression (10–16). Given the considerable clinical, cognitive, and genetic heterogeneity of ALS, there is an unmet need for early diagnostic biomarkers to aid patient stratification into specific phenotypes (17). Clinical trials of ALS continue to rely on survival, functional scores and respiratory measures as outcome measures despite the potential of candidate imaging markers (18).

Magnetic resonance imaging (MRI) not only contributed to the characterization of ALS-associated cerebral changes, it has also contributed important pathophysiological insights, such as the role of inflammation (19), patterns of spread (20, 21), inhibitory dysfunction (22, 23), and network-wise propagation (24, 25). In addition to describing unifying disease-associated signatures, imaging studies of ALS have gradually characterized the features of specific genotypes (26, 27), phenotypes (28, 29), the substrate of cognitive and extra-pyramidal impairments (30), as well as presymptomatic (31) and longitudinal changes (32). Despite the momentous advances however, the overwhelming majority of imaging studies in ALS remain cerebral, overlooking a disease-defining site of ALS pathology; the spinal cord (3).

## Spinal Cord Imaging

One of the key challenges of spinal cord imaging stems from its elongated dimensions, small cross-sectional area in the axial plane coupled with long sagittal and coronal expansion (33). Furthermore, the cord is surrounded by tissues that have very different magnetic susceptibility profiles and is it subject to both direct (cardiac and respiratory) and fluid-mediated [cerebrospinal fluid (CSF)] movement effects. The main challenges of quantitative spinal cord imaging include (i) partial volume effects, (ii) an inhomogeneous magnetic field environment, and (iii) physiological and patient motion (34).

## Methodological Challenges

### Partial Volume Effects

Partial volume refers to scenarios where different tissues contribute to the same voxel. In spinal cord imaging this occurs when a voxel is at the CSF/white matter, white matter/gray matter, CSF/vascular, white matter/vascular interfaces. Signals from different tissue densities with different amounts of spins contribute to the total MR signal in these voxels, which results in indistinct tissue-boundaries. Partial volume effects can be reduced by increasing the spatial resolution, but this in turn results in lower signal-to-noise (SNR) and contrast-to-noise ratios (CNR). Magnetic fields strengths of three or seven Tesla compared to conventional 1.5 Tesla platforms (35–38), higher number of phased-array coils with parallel imaging (35, 38, 39), and corrections for physiological motion improves spatial resolution, SNR, and CNR (35, 38, 39).

### Physiological and Patient Motion

Due to its proximity to the lungs and the heart, almost the entire spinal cord undergoes repetitive displacement due to respiration, CSF, and cardiac pulsation (40–43). The movement of the human spinal cord linearly increases caudally with distance from the head. The available literature suggest that physiological anterior-posterior (A-P) cord movement (0.60 ± 0.34 mm) exceeds those observed in superior-inferior (SI) (0.4 ± 0.1 mm) and right-left (RL) direction (0.17 ± 0.09 mm) (44, 45). Spinal imaging is also susceptible to movement artifacts from swallowing and patient movements during long MR acquisitions which can create ghosting artifacts (42, 46). By “gating” the acquisition, i.e., synchronizing with the respiratory or cardiac cycles, the effect of periodical movements can be significantly reduced (38, 39, 47). Motion artifacts can also be reduced using “saturation bands” that cover the esophagus, chest, and abdomen, by attenuating signals from moving structures so that it does not corrupt the signal from the spinal cord itself. Velocity compensating gradient sequences and signal averaging across multiple phases of motion can also be applied to minimize motion artifacts. Reducing acquisition time by using fast sequences, i.e., fast-spin-echo, parallel imaging that increases acquisition speed by factors from 1.5 to 3, i.e., SENSitivity Encoding/GeneRalized Autocalibration Partial Parallel Acquisition-type reconstructions, partial Fourier imaging, reducing the size of the phase-encoded direction, and decreasing the k-space matrix size effectively reduce both physiological and subject motion effects (48–53). MRI compatible cervical collars, which minimize involuntary neck movements, may also reduce movement artifacts (46). Co-registration of all data when dealing with multiple series acquisition, e.g., diffusion tensor imaging (DTI) and functional MRI (fMRI), can also be performed to limit the inconsistency in derived maps (54, 55).

### Inhomogeneous Magnetic Field Environment

The spinal canal is surrounded by bones, ligaments, disks, arteries, and venous plexi. Its proximity to the esophagus, mediastinum, and the lungs, each containing various amounts of air, create a challenging scanning environment. Adipose tissue, bone, and air have different magnetic susceptibility profiles, and respiration-induced B0 field fluctuations (43) also contribute to the inhomogeneity of the magnetic field around the spinal cord, resulting in geometric distortions and signal intensity loss (56). To some extent, these artifacts can be counteracted with “shimming.” Shimming aims at compensating for field inhomogeneities by creating an auxiliary magnetic field via shim coils (57). While shimming improves overall field homogeneity, it is limited to smooth variations across larger regions and cannot fully compensate for small, and localized field variations, such as those observed at cartilaginous discs between the vertebral bodies. Echo planar imaging sequences, such as DTI, are particularly sensitive to geometric distortions around vertebral disks. In addition to shimming, parallel imaging, and careful slices positioning may reduce magnetic field inhomogeneity, i.e., slices centered in the middle of each vertebral body and perpendicular to the spinal cord (38, 47, 58). The specific geometry of the magnetic field inhomogeneities should be considered in order to correct for its effect (59–61).

## Spinal Cord Imaging in ALS

The role of conventional spinal MRI in ALS is to rule of alternative structural, inflammatory or neoplastic pathologies which may result in a combination of upper and lower motor neuron involvement mimicking ALS (62). Compressive myelopathies and radiculopathies are relatively common and early, predominantly lower limb presentations of ALS are sometimes attributed to these radiological findings resulting in laminectomies and other invasive procedures (63, 64). Conventional, clinical spinal sequences are typically only qualitatively interpreted without specific measurements. The majority of clinical spinal scans in ALS are reported as normal, but non-specific signs such as high signal along the corticospinal tracts are occasionally observed on T2-weighted imaging (65–67).

In sharp contrast with clinical sequences, advanced quantitative spinal protocols allow for the detailed and quantitative characterization of spinal gray and white matter integrity (38, 47, 58, 68). These protocols provide high resolution, high SNR, and high CNR images compared to standard clinical sequences. Furthermore, purpose-designed spinal protocols are based on mathematical MR signal modeling (e.g., diffusion-based methods, quantitative magnetization transfer, and MR spectroscopy) and the derived outputs can be quantitatively interpreted to provide accurate, motion-corrected white, and gray matter metrics.

### Cord Morphometry

Gross axonal and gray matter loss have traditionally been estimated by measuring spinal cord cross-sectional areas at specific levels and interpreted as a proxy of atrophy in the context of reference normative values (69–72). The “cross-sectional approach” consists of estimating a mean cord cross-sectional area over a representative number of slices at a given vertebral level (70, 71, 73, 74), which can be relatively easily calculated from conventional MR sequences such as T1- or T2-weighted images. A variety of indexes, such as A-P dimension, L-R width, and radial distance can be derived from the cross-sectional area (CSA) approach. These measures reflect on different aspects of pathology, such as global vs. regional, lateral vs. anterior tissue loss, and are often interpreted as predominantly motor or sensory involvement (70, 75). More specific gray and white matter measures can be derived from higher resolution images followed by tissue-type segmentation methods (72, 76, 77). Novel quantitative approaches, such as tensor based morphometry and surface based-morphometry permit a more fine-grained characterization of cord topography and the definition of disease-associated signatures (74, 78). Recent studies demonstrated that spinal cord atrophy, especially gray matter atrophy, correlates with disability and disease progression and may be predictive of respiratory failure and of survival in ALS (58, 70, 72, 73, 79). The main findings of structural spinal cord studies are summarized in Table 1.

**Table 1:** Quantitative spinal imaging studies in ALS, ALS, amyotrophic lateral sclerosis; ALSFRS-r, the revised ALS functional scale; FA, fractional anisotropy; CSA, cross-sectional area; CST, corticospinal tract; FVC, force vital capacity; ihMT, inhomogeneous magnetization transfer; ihMTR, inhomogeneous magnetization transfer ratio; MD, mean diffusivity; MT, magnetization transfer; MTR, magnetization transfer ratio; MMT, manual muscle testing; SC, spinal cord; SOD1, superoxide dismutase 1 gene.

**Author year of publication (references)**	**Patient cohort *n***	**Controls *n***	**Spinal imaging technique**	**Spinal cord region evaluated**	**Main findings**
Valsasina et al. (80)	28 Sporadic ALS	20	CSA/DTI	Cervical spinal cord	Decreased FA and CSA decreased in ALS. Strong correlation between FA and the ALSFRS and moderate correlation between spinal and brain FA
Agosta et al. (81)	17/17 at baseline/follow-up (9 months) Sporadic ALS	20	CSA/DTI	Cervical spinal cord	Longitudinal FA, MD, and CSA changes detected. Brain CST diffusivity measurements are stable over time and do not correlate with cord measures
Nair et al. (82)	14 Sporadic ALS	15	DTI	C2-C6 vertebral levels	Reduced FA and RD in ALS. FA and RD correlate with finger and foot tapping rates. RD correlates with FVC and ALSFRS-R
Carew et al. (31)	23 sporadic ALS, 24 presymptomatic SOD1carriers	29	1H-MRS	C2 vertebral level	Reduced NAA/Cr and NAA/Myo ratios in both SOD1+ and sporadic ALS. Reduced Myo/Cr in SOD1+ subjects but not in sporadic ALS. Reduced NAA/Cho in sporadic ALS but not in SOD1+ subjects
Carew et al. (83)	14 Sporadic ALS	16	1H-MRS	C2 vertebral level	Reduced NAA/Cr and NAA/Myo ratios in ALS. NAA/Myo and NAA/Cho reductions correlate with FVC
Ikeda et al. (84)	19 Sporadic ALS	20	1H-MRS	C2 vertebral level	Reduced NAA/Cr and NAA/Myo ratios in ALS. NAA/Cr and NAA/Myo correlate with ALSFRS and FVC. NAA/Cr, NAA/m-Ins, and m-Ins/Cr are markedly altered in patients with C2 denervation and neurogenic changes
Cohen-Adad et al. (69)	27 sporadic ALS, 2 SOD1-linked familial ALS	21	CSA/DTI/MT	C2-T2 vertebral levels	Altered DTI and MT metrics in the lateral and dorsal columns. FA correlates with ALSFRS-r. Segmental cord atrophy is associated with disability. FA profile of the cervical cord is suggestive of retrograde CST degeneration i.e., “dying back”
Branco et al. (70)	25 Sporadic ALS	43	CSA	C2 vertebral level	Decreased CSA in ALS. CSA correlates with disease duration, ALSFRS-r, and ALS severity scale
El Mendili et al. (73)	29 at baseline, 14 at follow-up	–	CSA/DTI/MT	C2-T2 vertebral levels	CSA correlates with MMT. At follow-up, CSA predicts upper limb ALSFSR-R subscores, and FA predicts lower limb disability. CSA and MTR decrease between baseline and follow-up
Wang et al. (85)	24 Sporadic ALS	16	DTI	C2-C4 vertebral levels	CST FA and ADC changes in ALS. No difference in FA or ADC between patients with “definite” and “probable” ALS. No correlations between DTI parameters and modified Norris or ALSFRS-r scores
Iglesias et al. (86)	21 Sporadic ALS	21	DTI	Cervical spinal cord	Abnormal DTI metrics indicate decreased integrity of ascending sensory fibers. Significant correlation between DTI metrics and the depression of the peripheral afferent volley. The combination of SEP and DTI reveals sub-clinical sensory deficits in 85% ALS patients
Rasoanandrianina et al. (58)	10 Sporadic ALS	20	CSA/DTI/MT/ihMT	Cervical spinal cord	Spinal GM and WM atrophy in ALS. GM atrophy correlates with UMN scores. FA and MTR decrease in the CST. Axial diffusivity and ihMT decreased in the CST and dorsal columns. CSA correlates with the ALSFRS-r and spinal ALSFRS-R subscores. DTI and MT/ihMT metrics correlate with disease duration and MRC scores
de Albuquerque et al. (87)	27 at baseline, 27 at follow-up 8 months apart	27	CSA/DTI	C2 vertebral level	Longitudinal reduction in CSA. Cord area reduction correlates with change in ALSFRS-r
Querin et al. (79)	49 sporadic ALS	–	CSA/DTI/MT	C2-T2 vertebral levels	Spinal MRI parameters are more predictive of survival than clinical variables (ALSFRS-R, MMT, and disease duration)
Paquin et al. (72)	27 sporadic, 2 SOD1-linked familial ALS	22	CSA	C3-C6 vertebral levels	Spinal gray matter metrics are more sensitive to discriminate ALS patients from controls than overall cord CSA. Gray matter and spinal cord CSA correlates with ALSFRS-r and MMT arm scores. ALSFRS-r prediction improves when including a combination of gray and white matter CSA
Querin et al. (76)	60 sporadic ALS	45	CSA/DTI/MT	Cervical spinal cord	Random forest classification algorithm leads to good diagnostic performance distinguishing patients with ALS from controls with a sensitivity of 88% and specificity of 85%. The highest discrimination ability was achieved by evaluating RD, followed by FA, and CSA at the C5 spinal level
Piaggio et al. (88)	23 Sporadic ALS	18	CSA	Level of the Foramen magnum	Spinal cord area at the foramen magnum is significantly lower in ALS patients than in control subjects and is significantly correlated to ALSFRS-r. Spinal cord CSA at the foramen magnum correlates with disability in ALS independently of cerebral measures
Grolez et al. (89)	40 at baseline, 40 at follow-up 3 months apart	21	SC volume	Cervical spinal cord	Longitudinal change in cervical spinal cord volume is predictive of slow vital capacity decline and is also associated with survival

### Diffusion Weighted Imaging

Diffusion weighted imaging (DWI) relies on the evaluation of water diffusion in CNS tissues and is primarily used to characterize white matter integrity (90, 91). DWI-derived metrics, such as axial diffusivity (AD), mean diffusivity (MD), fractional anisotropy (FA), radial diffusivity (RD) enable the quantitative characterization of white matter integrity. Novel high-directional approaches, such as high-angular resolution diffusion imaging (92), q-ball imaging (93), diffusion kurtosis imaging (94), diffusion basis spectrum imaging (DBSI) (95) are particularly well-suited to assess the integrity of crossing-fibers (96, 97). Emerging diffusion techniques such as neurite orientation dispersion and density imaging (NODDI) (98) help to estimate the microstructural attributes of dendrites and axons (99). While in ALS NODDI has been primarily used in cerebral studies in ALS (100, 101), it also has been also piloted in spinal applications (90, 102). Specific DTI indices (AD, RD) have been associated with specific pathological processes, such as axonal (103, 104) vs. myelin-related (105, 106) degeneration, but this interpretation is likely to be simplistic, as DTI measures are affected by axonal density, axonal diameter, myelin thickness and fiber orientation, fiber coherence, and acquisition parameters. DTI has been extensively used to study cerebral changes in ALS and describe phenotype-associated (107), genotype-specific (27), presymptomatic (32), and longitudinal white matter changes in the brain (81). In contrast to the plethora of cerebral DTI studies, relatively few spinal DTI studies have been published in ALS to date (58, 69, 73, 80–82, 85). These have consistently highlighted both motor and sensory tract alterations (Table 1).

### Magnetization Transfer Imaging

Hydrogen nuclei linked to macromolecules such as the proteins and lipids of the myelin sheet have an extremely short T2 signal. While these macromolecules are not directly detectable by standard MRI sequences, magnetization transfer (MT) imaging enables the characterization of these structures. Macromolecular spins can be saturated using an off-resonance RF pulse, then the magnetization transfer between bound and free pools can be measured (108). Magnetization transfer occurs by means of cross relaxation processes, such as dipole-dipole interactions and chemical exchange. Magnetization transfer ratio (MTR) is calculated as the percentage difference of MT images with macromolecules signal saturation and one without. MTR enables inferences on myelin content, axonal count, and density as shown by three MS histological studies, and has been used extensively to assess demyelination, remyelination, and degeneration in MS (109–111). Conversely, relatively few studies have used cerebral MT imaging in ALS, and the majority of these focused on corticospinal tract alterations (112–115). Relatively few studies evaluated spinal MT changes in ALS, but they have shown progressive reduction overt time and correlation with muscle weakness (58, 69, 73). The key findings of spinal MT imaging studies in ALS and associated technical challenges are summarized in Tables 1, 2.

**Table 2:** The advantages and methodological challenges associated with specific spinal imaging techniques.

**Imaging technique**	**Advantages of specific techniques in ALS**	**Challenges and correction strategies**
Diffusion-weighted imaging	Evaluation of specific white matter bundles; motor; and sensory white matter tracts integrity. Availability of multiple derived diffusivity metrics reflecting on various histological aspects of white matter integrity; AD, MD, RD, FA. Emerging high angular resolution diffusion techniques to assess crossing fiber integrity. Derived metrics can be interpreted in comparative, longitudinal, correlation, and machine learning analyses	Motion artifacts: - Gating the acquisition (DWI, CSA, and volume estimation, fMRI, 1H-MRS) - Saturation bands (all modalities) - Velocity compensating gradient sequences (DWI) - Signal averaging across multiple phases of motion (DWI, fMRI, 1H-MRS) - Fast sequences (DWI) - MRI compatible cervical collar (DWI, CSA, and volume estimation, fMRI, 1H-MRS) - Co-registration of all data (DWI, fMRI) - Non-linear co-registration between T1 with and without magnetization transfer saturation pulse (MTR, ihMT) Magnetic field inhomogeneities: - Shimming (all modalities) - Parallel imaging (all modalities) - Corrections for gradients nonlinearity induced geometric distortion (DWI, MT, ihMT, CSA, and volume estimation, fMRI) - Corrections for breathing induced B0 field fluctuations (DWI, fMRI, CSA) Partial volume effect (all modalities) - Higher magnet field strength - Higher number of phased-array coils with parallel imaging - Multi-channel image acquisition - Limiting physiological motion
Magnetization transfer imaging	Evaluation of both white and gray matter integrity. Sensitive detection and measurement of demyelination. Derived metrics can be evaluated at individual and group-level statistical analyses	
Inhomogeneous magnetization transfer imaging	Applicability to both gray and white matter tissue components, superior sensitivity to detect demyelination	
Cross-sectional area and volume estimation	Automated segmentation pipelines enable the estimation of overall cord cross-sectional area and gray and white matter components separately. Gray matter components correlate with clinical and electrophysiological lower motor neuron (LMN) measures, therefore may be regarded an imaging proxy of LMN integrity	
1H-MR spectroscopyd	MRS provides a number of metrics which reflect on focal neuronal integrity (NAA), energy metabolism (Cr), membrane integrity (Cho), and glial function (Myo). MRS readily captures segmental metabolic alterations in symptomatic and presymptomatic ALS cohorts	
Functional MRI	As an emerging technique spinal fMRI has the potential to detect segmental cord activation during motor tasks and at rest	

*ALS, amyotrophic lateral sclerosis; ALSFRS-r, revised ALS functional scale; FA, fractional anisotropy; CST, corticospinal tract; FVC, force vital capacity; MD, mean diffusivity; MMT, manual muscle testing*.

### Inhomogeneous Magnetization Transfer Imaging

Inhomogeneous magnetization transfer (ihMT) imaging is a novel method (116, 117), which allows the unprecedented characterization of myelin integrity, by isolating key myelin components from the broader macromolecular pool. ihMT shows unparalleled potential to detect and quantify demyelination (118) and may be adapted to spinal applications. ihMT imaging has already been applied to ALS cohorts and demonstrated significant correlation with muscle strength and disability profiles (58).

### MR Spectroscopy

Magnetic resonance spectroscopy (MRS) is well-established, non-invasive imaging tool which provides neurochemical insights based on the concentration and relaxation profile of specific metabolites in cerebral and spinal tissues. MRS has been extensively used in cerebral studies of ALS (119), used to assess the therapeutic effect of Riluzole (120, 121), and also used to study brainstem metabolic alterations (122). Cross-sectional and longitudinal (123), single voxel and whole brain multi-voxel studies have both contributed to our understanding of ALS pathophysiology (124). The main targets of proton spectroscopy (1H-MRS) include the following metabolites; N-Acetyl Aspartate (NAA), creatine (Cr), choline (Cho), and myo-Inositol (Myo). These metabolites are typically associated with neuronal integrity/viability (NAA), tissue energy metabolism (Cr), membrane integrity (Cho), and glial function (Myo). (125). Relatively few studies have used 1H-MRS to characterize metabolic changes at the spinal level, and the majority of these studies focused on multiple sclerosis (126, 127) MRS however seems particularly applicable to ALS cohorts, where it promises the characterization of presymptomatic changes and by including both the upper and lower motor components of the motor system, it has led to particularly significant clinico-radiological correlations (31, 83, 84). For the contribution of MRS studies to ALS research and specific methodological considerations please see Tables 1, 2.

### Functional MRI

Functional MRI (fMRI) detects local variations in blood oxygenation level-dependent MR signal at rest and during activation paradigms (128). FMRI has been extensively applied to ALS cohorts to describe network changes and assess altered activation patterns when performing motor or cognitive tasks (129–131). Following decades of successful cerebral studies, the first spinal fMRI studies have now been published (55, 132). Emerging spinal cord fMRI studies in healthy controls provide proof of feasibility and the first studies using spinal fMRI in neurological conditions are underway (133).

## The Contribution of Spinal Imaging to ALS Research

### Evidence for Motor Involvement in ALS

Quantitative spinal MRI studies in ALS have consistently detected corticospinal tract and anterior horns degeneration and changes correlated with functional disability (36, 58, 80, 82, 85). Segmental spinal cord atrophy was not only linked to muscle weakness (58, 70, 88), but also to electrophysiological markers such as transracial magnetic stimulation (TMS) and motor evoked potentials (69). Two studies have demonstrated that both white and gray matter atrophy contributes to global cord atrophy in ALS (58, 72), but a recent study indicates that cord atrophy in ALS may be predominantly driven by anterior horn degeneration (72), confirming the role of spinal MRI as a putative LMN marker. DTI and MTR indices of the corticospinal tract (CST) correlated with TMS facilitation motor thresholds, a functional parameter that reflects pyramidal tract integrity.

### Longitudinal Spinal Cord Changes in ALS

In contrast to the plethora of longitudinal cerebral studies in ALS (21), relatively few longitudinal spinal studies are available to demonstrate that spinal MRI metrics can track subtle progressive changes over time (73, 81, 87, 89). These longitudinal studies captured decreasing CST MTR and progressive cord atrophy (73, 87) While some longitudinal studies also captured progressive DTI alterations (81), other studies did not (73). Some studies suggest that CSA estimates may be more reliable markers of longitudinal cord pathology than MTR or DTI metrics (73, 87). Progressive cord atrophy not only mirrors clinical progression, but early cervical cord atrophy is thought to predict respiratory dysfunction in ALS (89, 134). Furthermore, spinal MRI metrics may be superior predictive indicators of survival than clinical measures (79). Given the scarcity of longitudinal spinal imaging studies in ALS, it remains to be established which imaging metrics capture early ALS-associated changes, therefore may be used in diagnostic applications, and which metrics can track changes in the later stages making them suitable as monitoring markers.

### Evidence for Sensory Involvement in ALS

Several spinal MRI studies (58, 69) have captured dorsal column degeneration using DTI, MT, and ihMT imaging, and one study demonstrated progressive sensory tract degeneration over time (135). Dorsal column pathology can be detected relatively soon after symptoms onset, which suggests that sensory involvement is a core and relatively early feature of ALS. Combined spinal DTI and neurophysiology studies have also confirmed considerable sensory pathway degeneration in ALS patients without sensory symptoms (86). The combined MRI-neurophysiology approach revealed sub-clinical sensory deficits in 85% of ALS patients. These findings suggest that sensory dysfunction may have been underestimated by previous studies and that sensory afferent pathways may be affected early in the course of ALS and are important facets of ALS pathogenesis (69, 86). In contrast to longitudinal cerebral studies (4, 32), longitudinal spinal studies suggest that dorsal column metrics (73), and CST DTI indices (87) may be relatively constant (135).

### Evidence for Spinal Metabolic Alterations in ALS

1H-MRS studies in ALS have shown reduced NAA/Cr and NAA/Myo ratios at the C2 vertebral level (31, 83, 84). One spinal MRS study captured reduced NAA/Myo and NAA/Cr ratios in presymptomatic superoxide dismutase 1 gene (SOD1+) carriers (31). In addition to group-level differences in symptomatic and presymptomatic ALS cohorts, NAA/Myo and NAA/Cho reductions correlate with force vital capacity (FVC) and revised ALS functional scale (ALSFRS-r) and inversely correlated to the rates of decline (31, 83, 84).

## Future Directions

Existing spinal studies in ALS indicate that it is possible to detect disease-specific imaging signatures at a group level, and emerging machine-learning studies (76) have demonstrated that it may be possible to classify individual scans into “ALS” and “Healthy” groups. Despite the pioneering studies however, it is clear that spinal imaging lags behind cerebral imaging. Cerebral imaging has shown that phenotype and genotype specific patterns can be detected, multi-time point longitudinal studies have shown divergent rates of gray and white matter degeneration, studies have been validated by post mortem examination and robust multi-site studies have also been published (136). It is likely that improved coil designs with higher number of phased-array elements, new generation scanners with higher gradients optimized for advanced diffusion-weighted imaging, ultra-high filed platforms with superior spatial resolution, and SNR, spinal imaging will contribute unprecedented insights in ALS. It is conceivable that spinal imaging will contribute to the longstanding debate about dying back and dying forward, and ALS being a primarily spinal vs. cerebral disease. Spinal imaging provides a unique opportunity to appraise both lower and upper motor neuron degeneration. It is also likely that imaging sequences currently primarily used in cerebral imaging in ALS such as resting state fMRI, task-based fMRI, quantitative susceptibility weighted imaging, presymptomatic imaging, texture analyses, and post mortem imaging will filter down to spinal applications. Data-sharing initiatives, cross-platform harmonization, inclusion of upper motor neuron (UMN) and lower motor neuron (LMN) predominant ALS cohorts, correlations with advanced neurophysiological techniques are trends of ALS imaging which is likely to be adopted in spinal studies. One of the key ambitions of multiparametric spinal imaging is to overcome the methodological challenges of thoracic and lumbar imaging.

## Conclusions

The momentous advances in spinal imaging in ALS suggest the spinal metrics may soon be used as validated diagnostic, monitoring, and prognostic markers, contributing both to individualized patient care and pharmacological trials.

## Author Contributions

ME, GQ, PB, and P-FP contributed equally to the conceptualization, drafting, and revision of the manuscript.

### Conflict of Interest Statement

The authors declare that the research was conducted in the absence of any commercial or financial relationships that could be construed as a potential conflict of interest.

## Abbreviations

1H-MRS: proton spectroscopy
A-P: anterior-posterior
AD: axial diffusivity
ALS: Amyotrophic lateral sclerosis
ALSFRS-R: revised ALS functional scale
Cho: choline
CNR: contrast-to-noise ratio
Cr: creatine
CSA: cross-sectional area
CSF: cerebrospinal flood
CST: corticospinal tract
DTI: diffusion tensor imaging
FA: fractional anisotropy
fMRI: functional MRI
ihMT: Inhomogeneous magnetization transfer
LMN: lower motor neuron
MD: mean diffusivity
MRI: Magnetic resonance imaging
MRS: Magnetic resonance spectroscopy
MT: Magnetization transfer
MTR: Magnetization transfer ratio
Myo: myo-Inositol
NAA: N-Acetyl Aspartate
NODDI: neurite orientation dispersion and density imaging
RD: radial diffusivity
RL: right-left
SNR: signal-to-noise ratio
TMS: transracial magnetic stimulation
SOD1: superoxide dismutase 1 gene
SOD1+: presymptomatic superoxide dismutase 1 gene.
